# A Novel Gene Signature of Tripartite Motif Family for Predicting the Prognosis in Kidney Renal Clear Cell Carcinoma and Its Association With Immune Cell Infiltration

**DOI:** 10.3389/fonc.2022.840410

**Published:** 2022-03-17

**Authors:** Di Zheng, Yunlong Zhang, Yuqi Xia, Fan Cheng

**Affiliations:** Department of Urology, Renmin Hospital of Wuhan University, Wuhan, China

**Keywords:** tripartite motif family, KIRC, signature, prognosis, immune cell infiltration

## Abstract

Given the importance of tripartite motif (TRIM) proteins in diverse cellular biological processes and that their dysregulation contributes to cancer progression, we constructed a robust TRIM family signature to stratify patients with kidney renal clear cell carcinoma (KIRC). Transcriptomic profiles and corresponding clinical information of KIRC patients were obtained from The Cancer Genome Atlas (TCGA) and the International Cancer Genome Consortium (ICGC) databases. Prognosis-related TRIM family genes were screened and used to construct a novel TRIM family-based signature for the training cohort. The accuracy and generalizability of the prognostic signature were assessed in testing, entire, and external ICGC cohorts. We analyzed correlations among prognostic signatures, tumor immune microenvironment, and immune cell infiltration. The results of univariate Cox regression and Kaplan-Meier survival analyses revealed 27 TRIMs that were robustly associated with the prognosis of patients with KIRC. We applied Lasso regression and multivariate Cox regression analyses to develop a prognostic signature containing the *TRIM1*, *13*, *35*, *26*, *55*, *2*, *47*, and *27* genes to predict the survival of patients with KIRC. The accuracy and generalizability of this signature were confirmed in internal and external validation cohorts. We also constructed a predictive nomogram based on the signature and the clinicopathological characteristics of sex, age, and T and M status to aid clinical decision-making. We analyzed immune cell infiltration analysis and found that CD8 T cells, memory resting CD4 T cells, and M2 macrophages were the most enriched components in the KIRC tumor immune microenvironment. A higher level of immune infiltration by plasma cells, follicular helper T cells, and activated NK cells, and a lower level of immune infiltration by memory resting CD4 T cells, M1 and M2 macrophages, and resting dendritic cells were associated with higher risk scores. Overall, our eight-gene TRIM family signature has sufficient accuracy and generalizability for predicting the overall survival of patients with KIRC. Furthermore, this prognostic signature is associated with tumor immune status and distinct immune cell infiltrates in the tumor microenvironment.

## Introduction

Renal cell carcinoma (RCC) is the most common malignancy of the kidney. It arises from the renal tubule epithelium and accounts for ~ 90% of all renal tumors (1) and is the sixth most frequently diagnosed cancer in men worldwide, accounting for 5% of all oncological diagnoses each year (2). Based on its histological properties, RCC consists of kidney renal clear cell carcinoma (KIRC), kidney renal papillary cell carcinoma (KIRP), kidney chromophobe (KICH), and other relatively rare subtypes such as collecting duct carcinoma, medullary carcinoma, and other undefined subtypes (3). Epidemiological findings suggest that KIRC is the most common RCC subtype and accounts for 70% of all RCC diagnoses, followed by KIRP (10%–15%) and KICH (4%–6%) of RCC (4). Despite the rapid progress of diagnosis and treatments over the past two decades, RCC remains one of the most lethal malignancies of the urological system. Patients with localized RCC can be managed with surgery, whereas systemic therapy is the mainstay treatment for patients with relapsed or metastatic RCC (5). Tumor recurrence and metastasis are the main causes of RCC-related deaths, and 17% of patients already have distant metastasis by the time of diagnosis. Clinical factors, such as tumor grade and stage, are the main traditional predictors of overall survival (OS) for malignancies. However, the accuracy of prediction based on clinical factors is still poor in RCC owing to the complexity of inter-tumor and intra-tumor heterogeneity at the genomic level (6). Thus, to predict the prognosis of RCC patients remains challenging, and novel, reliable markers are needed to predict patient prognosis.

The tripartite motif (TRIM) superfamily comprises more than 80 genes (7). The proteins encoded by TRIMs have similar characteristic structures. The N-terminus of TRIM proteins is highly conserved and contains a tripartite motif comprising one RING domain, one or two B-box domains, and one coiled-coil domain, and C-terminal domains that are responsible for their diversity (8). Most TRIM proteins act as E3 ubiquitin ligases that regulate the degradation of target proteins, thus playing critical roles in cell proliferation, DNA damage and repair, and immune responses (7, 9, 10). Emerging evidence indicates that TRIMs are associated with the occurrence and development of cancers (11, 12) and might serve as diagnostic or prognostic biomarkers in various tumor types, including KIRC (13, 14).

Here, we comprehensively analyzed TRIM genes in KIRC based on datasets from The Cancer Genome Atlas (TCGA) and the International Cancer Genome Consortium (ICGC). Prognosis-related TRIM genes were identified and used to construct a robust prognostic signature in KIRC. The accuracy and specificity of the signature to predict the prognosis of patients with KIRC were validated in internal and external cohorts. We also analyzed associations between the prognostic signature and immune cell infiltration in patients with KIRC.

## Materials And Methods

### Data Acquisition and Processing

Transcriptome profiling data in fragment per kilobase method (FPKM) format of 539 KIRC tumor tissues and 72 non-tumor tissues were acquired from the TCGA (https://portal.gdc.cancer.gov/) database. The corresponding clinical information of KIRC patients were also obtained from the TCGA database. Additional gene expression matrix containing clinical information of KIRC patients were downloaded from the ICGC (https://dcc.icgc.org/projects) database and was regarded as the external validation cohort. Patient with missing clinical information were excluded for further analysis.

### Construction and Validation of the Prognostic Gene Signature of TRIM Family

We first identified prognosis-related genes of TRIM family by performing univariate Cox regression analysis and Kaplan-Meier survival analysis in TCGA KIRC cohort. Genes with *P*-value less than 0.05 in both analyses were considered as prognosis-related TRIM genes in KIRC. Subsequently, the TCGA KIRC cohort (entire cohort) was randomly classified into a training cohort and a testing cohort in a ratio of approximately 1:1. In training cohort, LASSO (Least absolute shrinkage and selection operator) regression analysis was conducted using *glmnet* package in R to prevent the occurrence of overfitting and obtain candidate genes. Then, stepwise multivariate Cox proportional hazards regression analysis was performed to calculate the corresponding coefficient and we ultimately developed an eight-gene prognostic signature of TRIM family in KIRC. The risk score of KIRC patients were calculated on the basis of linear combination of gene expression values and regression coefficients and the formula was as follow: $\text{Risk score}=\Sigma_{i}^{n}{\text{ Exp}}_{\text{i}}{\text{ Coe}}_{\text{i}}$ (Exp = gene expression value; Coe = regression coefficient). Later, patients in training cohort, testing cohort, entire cohort, and external validation cohort were categorized into high- and low-risk groups according to the median risk score value in training cohort. Kaplan-Meier survival analysis was applied to compare the overall survival between high- and low-risk groups using *survival* package in R. The sensitivity and specificity of the eight-gene signature were estimated by performing time-dependent receiver operating characteristic (ROC) curve analysis using *survivalROC* package.

### Functional Enrichment Analyses

Differentially expressed genes between high- and low-risk groups were identified in both TCGA and ICGC cohorts using *edgeR* package. Genes with |log2FC| >0.5 and FDR <0.01 were regarded as DEGs. Shared DEGs in the two cohorts were subjected to GO (Gene Ontology) and KEGG (Kyoto Encyclopedia of Genes and Genomes) enrichment analysis using *org.Hs.eg.db* and *clusterProfiler* package in R. Gene set enrichment analysis (GSEA) were performed to detect potential biological processes and cellular pathways enriched in low- or high-risk subgroups in TCGA and ICGC cohorts.

### Construction and Validation of a Predictive Nomogram

A nomogram comprising risk score and traditional prognosis-related clinical variables including age, gender, and T and M status was developed in TCGA cohort to quantitatively predict the prognosis of KIRC patients. The calibration curves were utilized to evaluate the sensitivity and accuracy of the nomogram in predicting the possibilities of 1-, 3- and 5-year overall survival for KIRC patients in TCGA and ICGC cohorts.

### Immune Cell Infiltration Analysis

The proportion of immune cell infiltration in each KIRC sample were estimated using the CIBERSORT algorithm. Briefly, normalized gene expression matrixes from the TCGA and ICGC cohorts were converted into 22 types of immune cell matrixes. After filtering with the criteria of *P*-value less than 0.05, the content of 22 types of immune cells between high- and low-risk groups in TCGA and ICGC cohorts were compared.

### Tissue Collection

A total of 22 frozen tissue samples including 11 kidney renal clear cell carcinoma tissues and 11 adjacent normal tissues were collected from patients with a histopathological diagnosis of KIRC undergoing surgery in Renmin hospital of Wuhan university between September 2020 and August 2021. These samples were harvest after resection and stored at -80°C. All the patients were given informed consent. The research was approved by the Ethics Committee of Renmin Hospital of Wuhan University.

### RNA Isolation and qRT-PCR

RNA was isolated using TRizol reagent (Invitrogen, Carlsbad, CA, United States) according to the manufacturer’s protocol, and was reverse transcribed into cDNA. Subsequently, quantitative real-time PCR (qRT-PCR) was performed to detect the relative gene expressions using the SYBR green PCR mix (vazyme, Nanjing, China). The primer sequences were list as follow: TRIM1, forward, 5’-ACTGGCCAGGCTAACTTCAT-3’ and reverse, 5’-TCGGGTAGGTTCACTGTTCC-3’; TRIM2, forward, 5’-TAACCAACCAAAAGGCCAGC-3’ and reverse, 5’-CGCTCATCTGCTTCTTCACC-3’; TRIM26, forward, 5’-ACCCATTGCTCGAGTGGTTA-3’ and reverse, 5’-ACTTCCCAGTAGACCTTGCC-3’; TRIM13, forward, 5’-AGCAAGATTCCCTGGAGCTT-3’ and reverse, 5’-TGCACACAAATTCTGCCACA-3’; TRIM27, forward, 5’-TGGATTCTGGGCAGTGTCTT-3’ and reverse, 5’-TCCCTCCCGAGTAACTCAGA-3’; TRIM55, forward, 5’-TACAAGCAGGAGTCCACCAG-3’ and reverse, 5’-CTGATCACTCCCTGGACTCG-3’; TRIM47, forward, 5’-AATCATCCCAAGCTGTCCGT-3’ and reverse, 5’-TAGGACACAGCACCCTCTTG-3’; TRIM35, forward, 5’-GCTTCGCGAGTTCTTGAGAG-3’ and reverse, 5’-GACATCGATAAGCATGCCGG-3’; GAPDH, forward, 5’-CTGAGTACGTCGTGGAGTCC-3’ and reverse, 5’-GTCTTCTGGGTGGCAGTGAT-3’;

### Statistical Analysis

All the statistical analyses were performed using R software (version 4.1.0). Kaplan-Meier survival curve analysis and a two-sided log-rank test was applied to compare overall survival between high- and low-risk groups. The Wilcoxon rank-sum test was employed to compare differences in the proportion of immune cell infiltration between two groups. A *P*-value less than 0.05 was regarded as statistically significant.

## Results

### Identification of Prognosis-Related TRIM Family Genes in KIRC and Construction of a Protein-Protein Interaction Network

**Figure 1:**
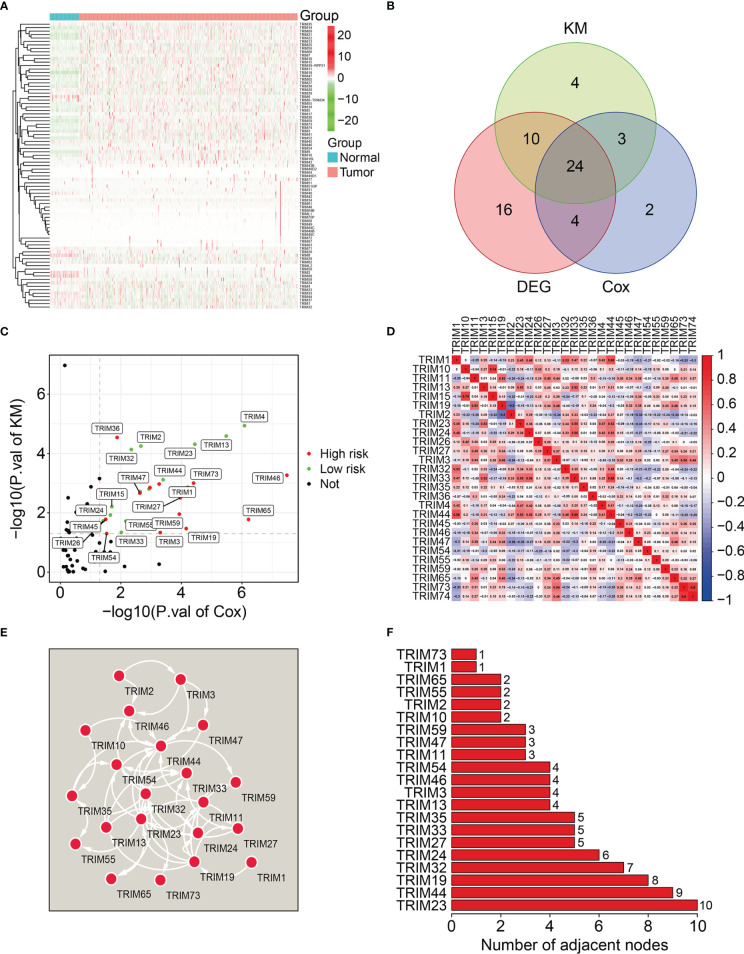
Identification of prognosis-related TRIMs in KIRC and construction of a protein-protein interaction network. **(A)** The expression profile of TRIMs in tumor tissues and adjacent normal tissues. **(B)** Venn plot showing the differentially expressed TRIMs between KIRC tissues and adjacent non-tumor tissues, and prognosis-related TRIMs. **(C)** Volcano plot showing the prognosis-related TRIMs. **(D)** Correlation heatmap of the 27 prognosis-related TRIMs. **(E)** Protein-protein interaction network of the 27 prognosis-related TRIMs. **(F)** Hub genes in the PPI network.

**Figure 1A** shows the expression profiles of TRIMs in KIRC tumor tissues and adjacent normal tissues. Among 83 genes in the TRIM family, 54 were differentially expressed between tumor and adjacent normal tissues (**Figure 1B**). Moreover, the findings of univariate Cox regression and Kaplan-Meier survival analyses revealed that 27 genes were robustly associated with the prognosis of patients with KIRC (**Figure 1B**). Fourteen of these 27 genes were protective (HR < 1) and 13 were risk factors (HR>1) for KIRC (**Figure 1C**). **Figure 1D** shows correlations among the 27 prognosis-related TRIMs. A protein-protein interaction network of these genes was constructed using STRING (https://string-db.org/) and visualized using Cytoscape (**Figure 1E**). Hub gene analysis suggested that TRIM23, 44, and 19 were the top three genes in a PPI network (**Figure 1F**).

### Construction of a Novel Signature Comprising Eight TRIM Genes in the Training Cohort

**Figure 2:**
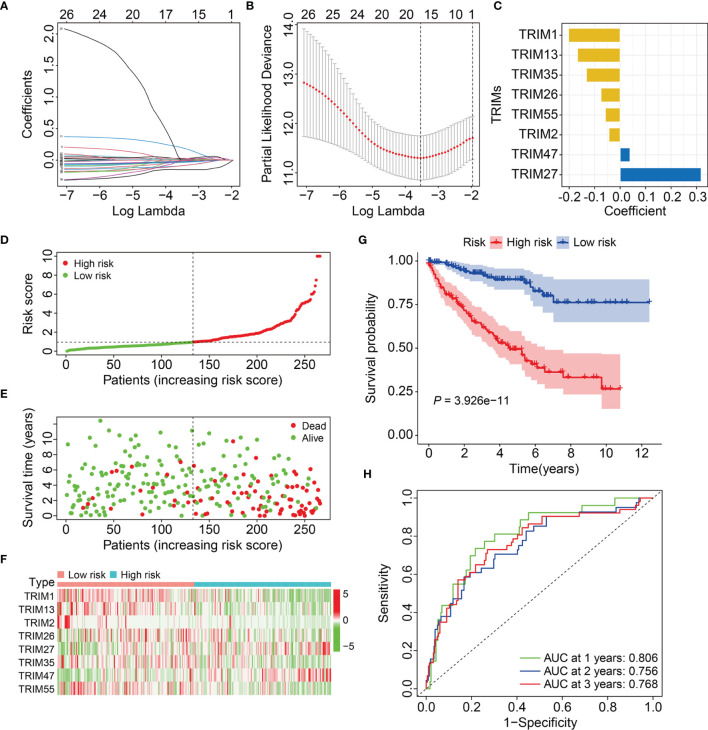
Construction of a novel eight-gene signature of TRIM family in the training cohort. **(A)** Lasso coefficients profiles of the prognosis-related TRIMs. **(B)** The association between deviance and log (lambda). **(C)** The coefficients of the eight TRIM genes. **(D)** Risk score distribution of KIRC patients in training cohort. **(E)** The distribution of survival time and survival status of KIRC patients in training cohort. **(F)** The expression profile of the eight TRIM genes. **(G)** Kaplan-Meier survival analysis in the training cohort. **(H)** Time-dependent ROC curve for 1-, 2-, and 3-year predictions in training cohort.

We analyzed the 27 prognosis-related TRIMs using Lasso regression followed by multivariate Cox regression in the training cohort (**Figures 2A, B**). We ultimately developed a prognostic signature of eight genes namely, TRIM1, 13, 35, 26, 55, 2, 47, and 7 to predict the survival of patients with KIRC. **Figure 2C** and **Table 1** show the coefficients and details of these eight genes. Risk scores for each patient in the training cohort were calculated on the expression and coefficients of the eight genes as *TRIM1* × (-0.202) + *TRIM13* × (-0.167) + *TRIM2*× (-0.044) + *TRIM35*× (-0.133) +*TRIM26* × (-0.075) +*TRIM55*× (-0.058) + *TRIM47* × 0.039 +*TRIM27* × 0.318.

**Table 1:** Details of the eight TRIM genes in the prognostic signature.

Gene Name	Coefficient	HR	HR.95L	HR.95H	*P*
*TRIM1*	-0.202	0.817	0.656	1.017	0.071
*TRIM13*	-0.167	0.846	0.713	1.003	0.055
*TRIM2*	-0.044	0.957	0.920	0.996	0.031
*TRIM26*	-0.075	0.928	0.874	0.985	0.014
*TRIM27*	0.318	1.374	1.172	1.612	0.000
*TRIM25*	-0.133	0.876	0.756	1.015	0.077
*TRIM47*	0.039	1.040	1.007	1.074	0.018
*TRIM55*	-0.058	0.943	0.892	0.998	0.043

Patients in the training cohort were allocated to high- or low-risk groups based on their median risk scores (**Figure 2D**). **Figure 2E** shows the survival status and survival duration of patients in the training cohort. The results suggested that patients in the low-risk group tended to have a lower death rate and prolonged survival. **Figure 2F** shows the expression profiles of the eight genes in the high- and low-risk groups. The results indicated that compared to those in the low-risk group, more *TRIM47* and *27* and less *TRIM1*, *13*, *35*, *26*, *55*, and *2* were expressed in the high-risk group. Kaplan-Meier curves revealed a better OS for patients in the low-risk group compared to that for patients in the high-risk group (**Figure 2G**). We evaluated the sensitivity and specificity of the eight-gene signature for predicting the prognosis of patients with KIRC using time-dependent receiver operator characteristics (ROC) curves. The areas under the ROC curves (AUCs) for 1-, 2-, and 3-year survival were 0.806, 0.756, and 0.768, respectively, indicating the accuracy of the prognostic signature (**Figure 2H**).

### Validation of Eight-Gene Signature in Internal and External Cohorts

**Figure 3:**
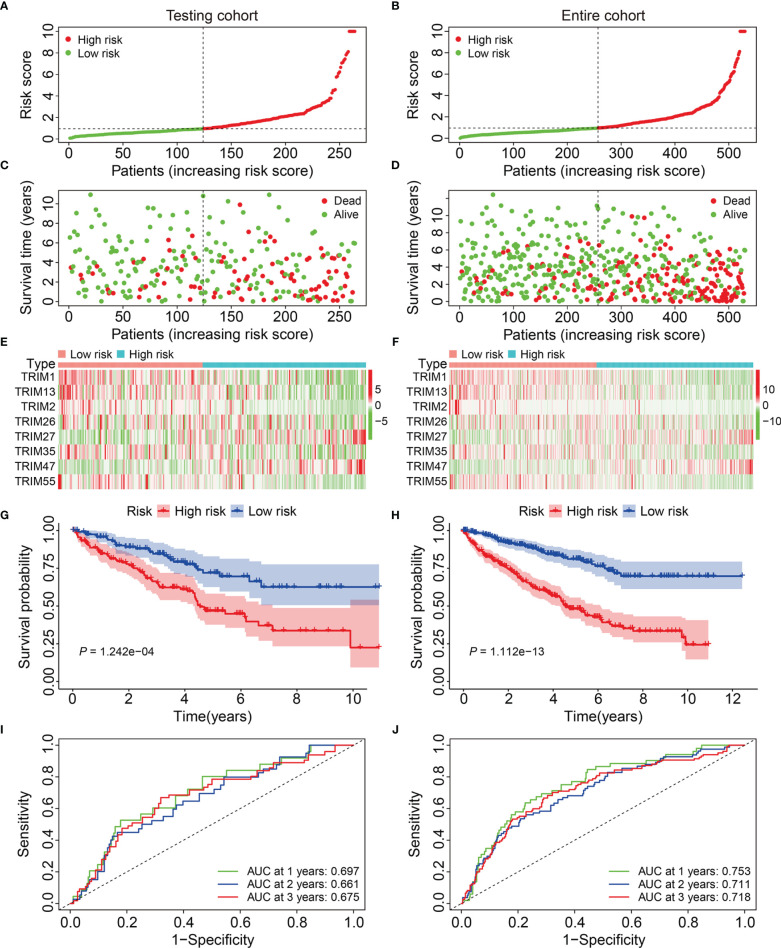
Validation of the eight-gene signature of TRIM family in the internal validation cohorts. **(A, B)** Risk score distribution of KIRC patients in testing and entire cohorts. **(C, D)** The distribution of survival time and survival status of KIRC patients in testing and entire cohorts. **(E, F)** The expression profile of the eight TRIM genes in testing and entire cohorts. **(G, H)** Kaplan-Meier survival analysis in testing and entire cohorts. **(I, J)** Time-dependent ROC curve for 1-, 2-, and 3-year predictions in testing and entire cohorts.

We calculated risk scores of the patients in the testing and entire cohorts using the same algorithm to validate the predictive ability of the eight-gene signature. Patients were allocated to high- or low-risk groups based on the median risk score in the training cohort (**Figures 3A, B**). The death rates were higher and survival was shorter in the high-risk group (**Figures 3C, D**). **Figures 3E, F** show the expression profiles of the eight genes in the test and entire cohorts. Kaplan-Meier curves revealed significantly better OS in the low-risk group compared to that in the high-risk group (**Figures 3G, H**). The AUCs for 1-, 2-, and 3-year OS were 0.697, 0.661, and 0.675, respectively in the test cohort (**Figure 3I**), and 0.753, 0.711, and 0.718, respectively, in the entire cohort (**Figure 3J**). These results showed that the signature of the eight TRIM genes has a strong prognostic value in both cohorts.

**Figure 4:**
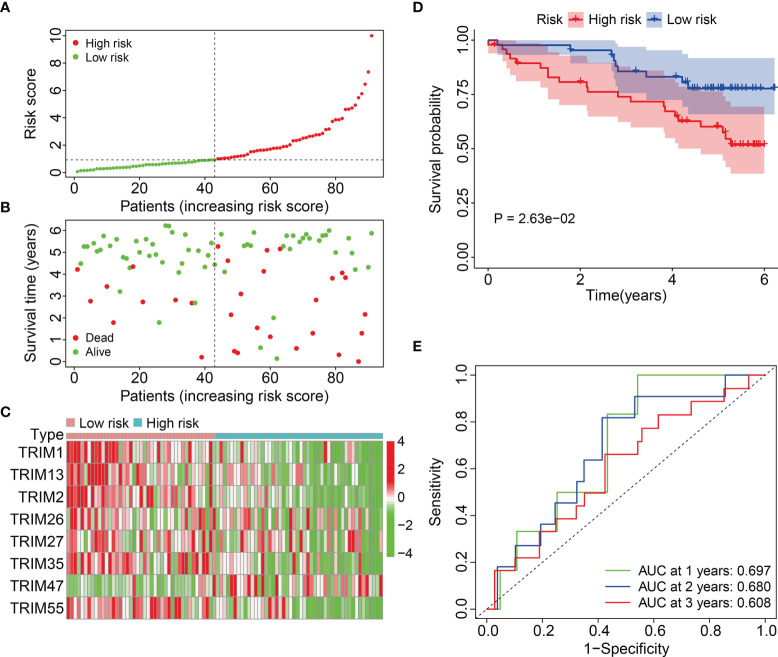
Validation of the eight-gene signature of TRIM family in external ICGC cohort. **(A)** Risk score distribution of KIRC patients in ICGC cohort. **(B)** The distribution of survival time and survival status of KIRC patients in ICGC cohort. **(C)** The expression profile of the eight TRIM genes in ICGC cohort. **(D)** Kaplan-Meier survival analysis in ICGC cohort. **(E)** Time-dependent ROC curve for 1-, 2-, and 3-year predictions in ICGC cohort.

We calculated the risk scores and set the median in the training cohort as the cut-off to stratify patients into high- and low-risk groups and further evaluate the generalizability and accuracy of signature (**Figure 4A**). **Figure 4B** shows a higher death rate among patients with KIRC in the high-risk group compared to that among patients in the low-risk group. **Figure 4C** shows the expression profiles of eight genes in the high- and low-risk groups. Kaplan-Meier curves indicated a significantly worse OS for patients in the high-risk group compared to that in the low-risk group (**Figure 4D**). The AUCs of the 1-, 2-, and 3-year OS in the external cohort were 0.697, 0.680, and 0.608, respectively (**Figure 4E**). Overall, these findings indicated that our eight-gene prognostic signature of the TRIM family had robust and stable prognostic ability.

### Stratification Analysis of Eight-Gene Prognostic Signature Based on Clinicopathological Features

**Figure 5:**
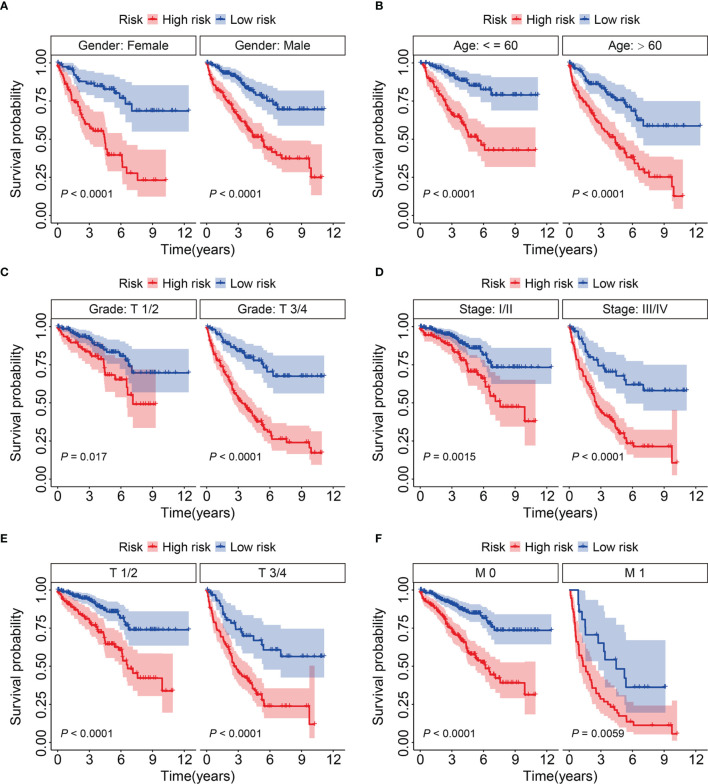
KM survival analysis of subgroups stratified by gender **(A)**, age **(B)**, grade **(C)**, stage **(D)**, and T and M status **(E, F)**.

We further assessed the predictive power of the eight-gene prognostic signature as follows. We assigned patients with KIRC in the entire cohort into subgroups based on the clinicopathological features of sex (female *vs.* male), age (≤ 60 *vs.* > 60 y), grade (T1/2 *vs.* T3/4), stage (I/II *vs.* III/IV), T (1/2 *vs.* T3/4), and M stage (M0 *vs.* M1). Compared to that in the high-risk group with different clinicopathological features, Kaplan-Meier curves (**Figures 5A**) indicated a better OS among patients with KIRC in the low-risk group.

### Prognostic Independence of the Signature and Construction of a Nomogram for KIRC

**Figure 6:**
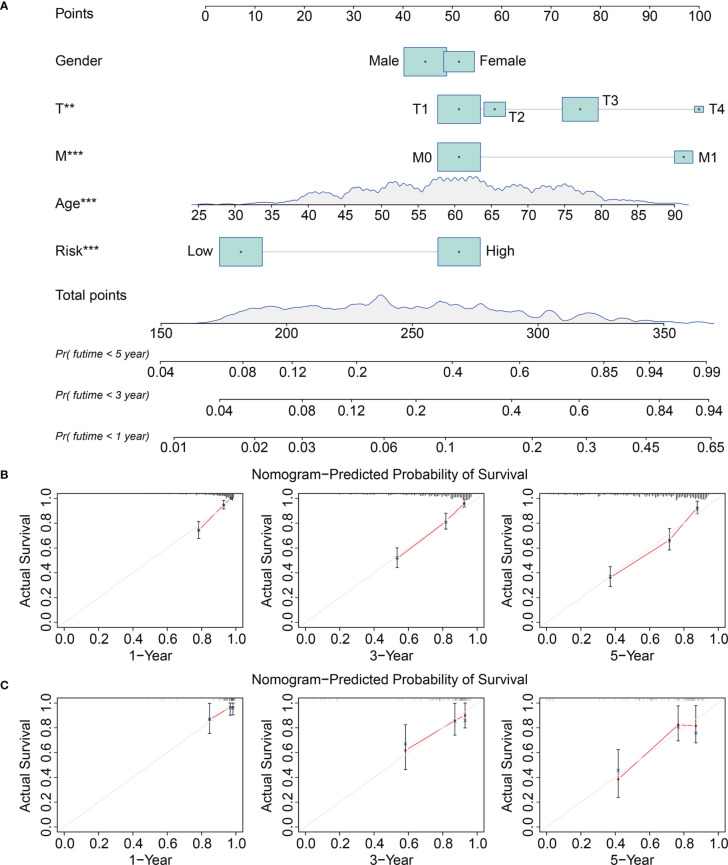
Construction and validation of a nomogram in TCGA and ICGC KIRC cohorts. **(A)** The nomogram comprising clinicopathological factors including gender, age, T and M status, and risk score based on the eight-TRIM gene signature. **(B, C)** The calibration curves displayed the concordance between predicted and observed 1-, 3-, and 5-year overall survival in TCGA **(B)** and ICGC **(C)** KIRC cohorts.

We analyzed the entire TCGA cohort using univariate and multivariate Cox regression to determine the independence of the eight-gene signature as a risk factor for KIRC. The results suggested that the risk score and the clinicopathological characteristics of age, grade, and T stage, were independent risk factors for patients with KIRC (**Table 2**). We then developed a prognostic nomogram to help clinicians decide how to treat or mange individual patients with KIRC. Risk scores, gender, age, T, and M stages in TCGA and ICGC cohorts were included in the nomogram (**Figure 6A**). Calibration curves for 1-, 3-, and 5-year survival predicted by the nomogram confirmed its predictive accuracy for KIRC in both TCGA and ICGC cohorts (**Figures 6B, C** **)**.

**Table 2:** Univariable and multivariable analysis of the signature based on TRIM genes and clinical factors in the TCGA cohort.

Variables	Univariable analysis				Multivariable analysis			
	HR	95% CI of HR		*P*	HR	95% CI of HR		P
		Lower	Upper			Lower	Upper	
Age (≤60 vs >60)	1.7879	1.3093	2.4414	0.003	1.6160	1.1758	2.2212	0.0031
Gender (Female vs Male)	0.9300	0.6789	1.2739	0.6513	0.9306	0.6729	1.2870	0.6637
Grade (I/II vs III/IV)	2.5928	1.8373	3.6589	0.0000	1.7297	1.1930	2.5078	0.0038
Stage (I/II vs III/IV)	3.6100	2.6180	4.9778	0.0000	1.9827	0.9541	4.1201	0.0667
T (T 1/2 vs T 3/4)	3.0027	2.2054	4.0881	0.0000	0.9456	0.5043	1.7731	0.8616
M (M0 vs M1)	4.2047	3.0699	5.7589	0.0000	2.4468	1.6546	3.6182	0.0000
Risk (High vs Low)	1.0416	1.0186	1.0651	0.0000	1.0349	1.0064	1.0641	0.0000

### Functions and Pathways Correlated With the Signature

**Figure 7:**
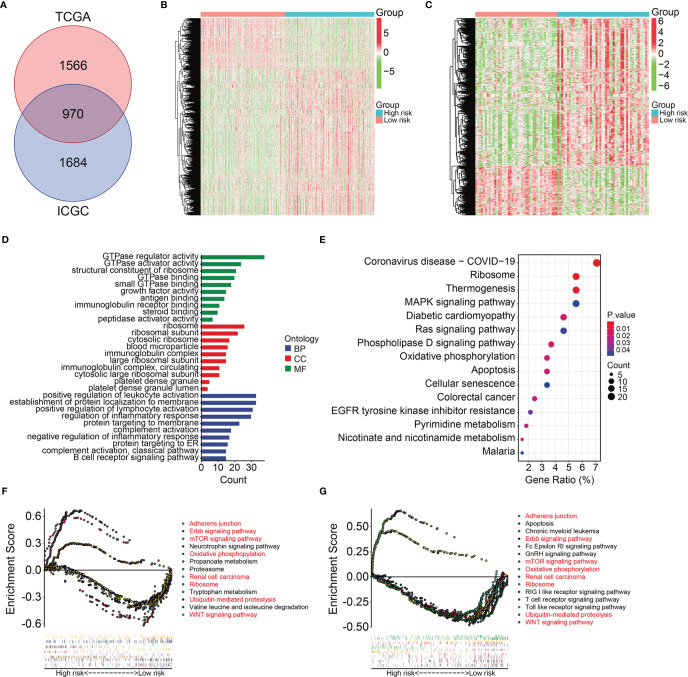
Identification of risk-related differential expressed genes and functional enrichment analysis. **(A)** Venn plot exhibiting differentially expressed genes between high- and low-risk groups in TCGA and ICGC cohorts. **(B–C)** The expression profiles of the differentially expressed genes in TCGA and ICGC cohorts. **(D)** GO enrichment analysis of the differentially expressed genes. **(E)** KEGG enrichment analysis of the differentially expressed genes. **(F, G)** Gene set enrichment analysis in TCGA and ICGC cohorts.

We further investigated the potential function of the signature using *limma* filtration and identified 970 shared differentially expressed genes (DEGs) between the high- and low-risk groups in TCGA and ICGC cohorts (**Figure 7A**). **Figures 7B, C** show expression profiles of these DEGs in TCGA and ICGC cohorts. Gene Ontology (GO) and Kyoto Encyclopedia of Genes and Genomes (KEGG) analyses of the 970 DEGs revealed significantly enriched DEGs in the biological processes of positive regulation of leukocyte activation, establishment of protein localization to the membrane, positive regulation of lymphocyte activation, regulation of inflammatory response, and complement activation. Ribosomes, ribosomal subunits, and cytosolic ribosomes were the three most enriched cellular components. We found that DEGs were particularly enriched in the molecular functions of GTPase regulator activity, GTPase activator activity, structural constituents of ribosomes, GTPase binding, and small GTPase binding (**Figure 7D**) and in the KEGG MAPK, Ras, phospholipase D signaling, ribosome, and oxidative phosphorylation pathways (**Figure 7E**). Gene set enrichment was compared between the high- and low-risk groups in TCGA and ICGC gene expression matrices. Oxidative phosphorylation and ribosome pathways were significantly enriched in the high-risk group in both TCGA and ICGC gene expression matrices. In contrast, adherens junction, ErbB signaling, mTOR signaling, WNT signaling renal cell carcinoma, and ubiquitin-mediated proteolysis pathways were enriched in the low-risk group (**Figures 7F, G** **)**.

### The Signature Correlated With Tumor Immune Cell Infiltration

**Figure 8:**
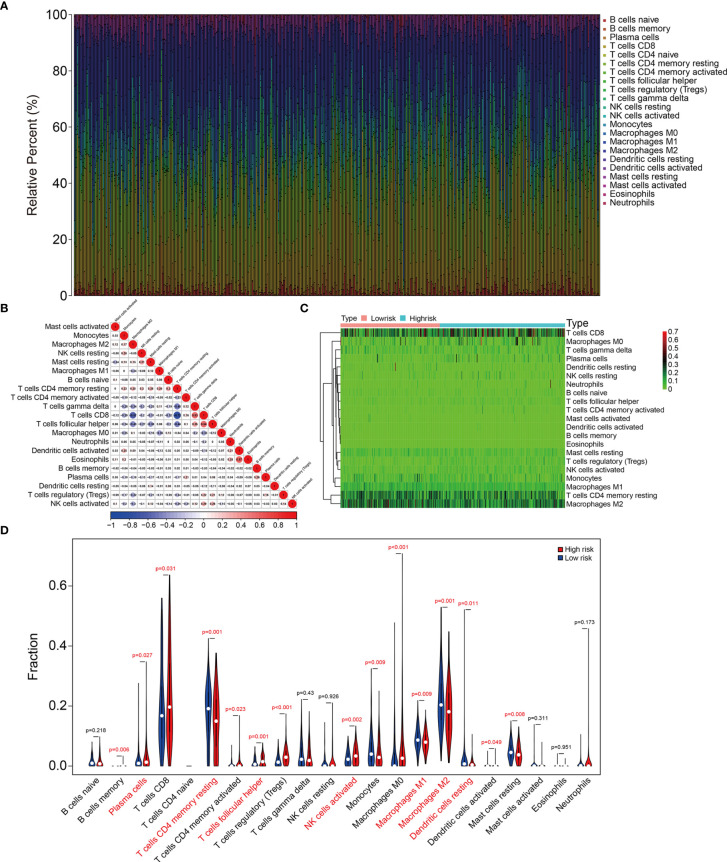
The signature was correlated with tumor immune cell infiltration in TCGA KIRC cohort. **(A)** Stacked bar chart showing the abundance of 22 immune cell types in each KIRC sample of the TCGA cohort. **(B)** The correlation heatmap of the infiltrating immune cells in the TCGA cohort. **(C, D)** Heatmap and violin plot exhibiting immune cell infiltrates in KIRC patients at high- and low-risk groups.

We further explored the association between signature and tumor immune cell infiltration in KIRC. **Figure 8A** and **Supplementary Figure 1A** show the proportions of 22 types of infiltrative immune cells in each KIRC sample in TCGA and ICGC cohorts. The three most enriched components in the tumor immune microenvironment were CD8 T cells, memory resting CD4 T cells, and M2 macrophages. **Figure 8B** and **Supplementary Figure 1B** show correlations among these cells in KIRC samples. We compared the content of tumor immune cell infiltration between the high- and low-risk groups of KIRC samples from TCGA and ICGC cohorts. **Figures 8C, D** and **Supplementary Figures 1C, D** show higher proportions of plasma cells, follicular helper T cells, and activated NK cells and lower proportions of memory resting CD4 T cells, resting dendritic cells, and M1 and M2 macrophages in the high-risk group. These findings suggested that the tumor immune microenvironment was closely associated with the prognosis of patients with KIRC.

### Expression and Kaplan-Meier Survival Analysis of the Eight TRIM Family Genes

**Figure 9:**
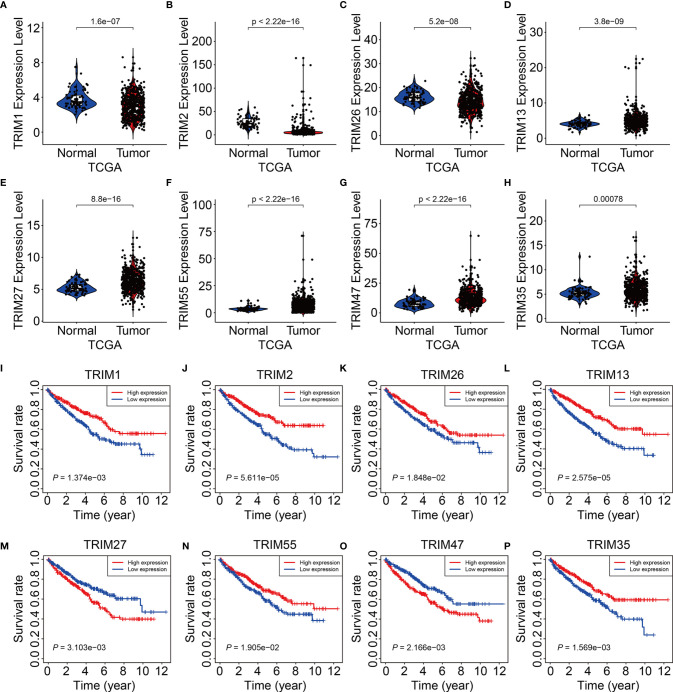
Expression and survival analysis of the eight genes of TRIM family. **(A–H)** The expression levels of *TRIM1*, *TRIM2*, *TRIM26*, *TRIM13*, *TRIM27*, *TRIM55*, *TRIM47*, and *TRIM35* in KIRC tumor tissues and adjacent normal tissues. **(I–P)** Kaplan-Meier curve analyses of the eight genes in TCGA KIRC cohort.

**Figure 10:**
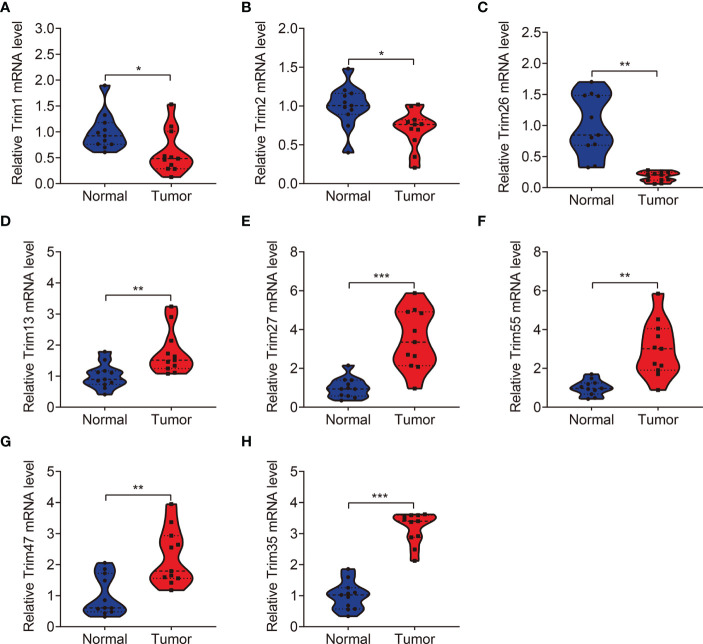
qRT-PCR was employed to detect the expression levels of *TRIM1*** (A)**, *TRIM2*** (B)**, *TRIM26*** (C)**, *TRIM13*** (D)**, *TRIM27*** (E)**, *TRIM55*** (F)**, *TRIM47*** (G)**, and *TRIM35* **(H)** in clinical KIRC samples. *P < 0.05, **P < 0.01, ***P < 0.001.

We analyzed expression of the eight TRIM genes and applied Kaplan-Meier curves to determine the survival of patients with KIRC derived from the public database and our experimental validations. Compared to that in adjacent normal tissues, we found significantly lower *TRIM1*, *2*, and *26* (**Figures 9A–C** and **Figures 10A–C**) and significantly higher *TRIM13*, *27*, *55*, *47*, and *35* expressions in KIRC tumor (**Figures 9D–H** and **Figures 10D–H**). Kaplan-Meier curves associated lower *TRIM1*, *2*, *26, 13*, *55*, and 3*5* expressions with a poorer prognosis of KIRC (**Figures 9I–L, N, P**), and lower *TRIM27* and *TRIM47* expression with a better OS (**Figures 9M, O**).

## Discussion

With the advancement of high-throughput sequencing and its application to cancer research, numerous prognostic biomarkers in diverse tumor types have been identified through bioinformatics analyses (15, 16). Recent reanalysis of datasets in public databases has provided an avenue to identify potential therapeutic targets, diagnostic or prognostic biomarkers, and predictor of response to therapy. An integrated analysis of glycolysis-related genes in clear cell renal cell carcinoma (ccRCC) by Lv et al. using TCGA and GEO datasets resulted in the construction of a seven-gene signature that predicted OS in ccRCC (17). The accuracy and generalizability of a prognostic signature for ccRCC created by Wu et al. based on ubiquitin-related genes has been verified in an independent dataset derived from the ArrayExpress database (18). Verbiest et al. found that the ccRCC molecular subtypes (ccrcc1 to 4), established based on unsupervised clustering of whole genome mRNA-expression data (19), could identify molecularly different patient populations and selected patients for systemic therapies, and they were predictive of sunitinib or pazopanib response in metastatic ccRCC (20, 21). These findings have provided new insights into the development of novel prognostic biomarker in KIRC.

The TRIM proteins play vital roles in various biological processes, and changes in them are involved in diverse pathological conditions, such as neurodegenerative diseases, immune-related diseases, and cancers (22–25). Dysregulated TRIM protein tumors are oncogenic or function as tumor suppressors (26–28). Many TRIMs are aberrantly expressed in RCC and might serve as prognostic biomarkers of RCC. For example, decreased TRIM2 expression in ccRCC, than that in adjacent normal renal tissues, is associated with a poor prognosis for patients with ccRCC (29). In contrast, increased TRIM44 expression is significantly associated with a worse OS in RCC (30). Abnormal TRIM expression contributes to the malignant biological behavior of cancer cells through diverse mechanisms. The mRNA and protein levels of TRIM47 were higher in human renal cancer tissues than those in paired normal adjacent tissues. Functionally, TRIM47 is an oncogene in RCC, and its overexpression accelerates cell proliferation and invasion by promoting P53 ubiquitination and degradation (14). TRIM37 is dysregulated in RCC, and its increased expression is associated with aggressive neoplastic phenotypes and poorer survival. Mechanistically, TRIM37 promotes the malignant progression of RCC *via* TGF-β1/SMAD signaling through its direct mediation by ubiquitinating-H2A modifications (31). Taken together, these findings indicate that TRIM proteins are associated with the occurrence and development of RCC and are potential biomarkers for prognostic prediction.

Given their important roles in RCC, our integrated analysis of TRIMs in KIRC, which is the most common RCC subtype, revealed that 54 (65%) of 83 TRIM family genes were differentially expressed between KIRC tissues and adjacent non-tumor tissues, further indicating the vital role of TRIMs in KIRC. Moreover, the results of univariate Cox regression and Kaplan-Meier curves showed that 27 TRIMs were robustly related to the prognosis of patients with KIRC. This prompted us to develop an eight-gene prognostic signature and further stratify patients into high- and low-risk groups. Compared to that in the low-risk group, the findings of Kaplan-Meier curves of all internal and external cohorts showed worse OS of patients in the high-risk group. We stratified patients into diverse subgroups based on the clinicopathological features of sex, age, grade, stage, and T and M status. Kaplan-Meier curves showed that survival was consistently worse in the high-risk groups compared to that in the low-risk groups in all subgroups. We verified the sensitivity and specificity of our prognostic signature based on TRIMs using time-dependent ROC analysis. Taken together, our findings showed that our TRIM-based signature is accurate and clinically useful for predicting the prognoses of patients with KIRC. Former research by Wu et al. analyzed the expression, mutations, and prognosis of 13 tumor stem cell associated TRIM genes (*TRIM6*, *TRIM8*, *TRIM11*, *TRIM14*, *TRIM16*, *PML*, *TRIM21*, *TRIM24*, *TRIM25*, *TRIM27*, *TRIM28*, *TRIM32*, and *TRIM71*) in KIRC, and they utilized these TRIM genes to construct a risk model and confirmed its prediction performance in internal but not in external KIRC cohort (23). Here, the accuracy of our TRIM-based signature was verified in both internal and external cohorts, suggesting the generalizability of the prognostic signature. Moreover, we constructed a prognostic nomogram based on risk scores, sex, age, and T and M stages. Our nomogram was helpful for clinical decision-making and personalizing the management of KIRC patients. To further determine the potential function of the eight-gene signature of the TRIM family, we identified DEGs between the high- and low-risk groups and analyzed functional enrichment. We found that immune-related biological processes were significantly enriched. Gene set enrichment analysis between the high- and low-risk groups revealed that tumor-related pathways, including adherens junction, ErbB, mTOR and WNT signaling pathways, renal cell carcinoma, and ubiquitin-mediated proteolysis were obviously enriched. We assessed the association of the eight-gene signature with tumor immune cell infiltration in KIRC and found that the high- and low-risk groups differed in terms of immune status and distinctive immune cell proportions. The importance of infiltrated immune cell for immunotherapy and prognosis had been recognized. The infiltrated B cells played role in the response to immune checkpoint blockade treatment (32). In localized ccRCC, a subgroup of patients whose tumors were infiltrated with polyclonal and poorly cytotoxic CD8+PD-1+ Tim-3+Lag-3+ cells and CD4+ICOS+ T cells had a very high risk of early disease progression (33). In KIRC, a higher level of infiltration memory resting CD4 T cells and resting dendritic cells was associated with improved outcome (34), and we found lower proportions of memory resting CD4 T cells and resting dendritic cells in high-risk group, which was consistent with predictive performance of these infiltrated immune cells in KIRC. Thus, our TRIM-based signature was closely associated with tumor immune cell infiltration and might be utilized to predict the response to immunotherapy, and TRIM genes might participate in this process. Former research reported that some of the TRIMs were interferon (IFN)-inducible proteins. For example, the expression of TRIM22 was increased during T lymphocyte activation with IL-15 (35). In tumor, IL-15 was associated with lymphocyte infiltration in the micro-environment (36), and we might speculate that TRIM22 mediated this process and further exploration awaits.

The signature comprised *TRIM1*, *13*, *35*, *26*, *55*, *2*, *47*, and *27* genes. Among these, the expression of *TRIM1*, *2*, and *26* was significantly lower in KIRC tumor, compared to that in adjacent normal tissues. This predicted a poorer prognosis and suggested a tumor-suppressive effect in patients with KIRC. However, the role of *TRIM1* remains unclear, whereas *TRIM2* plays dual roles by acting as an oncogene or as a tumor suppressor (37–39). In ccRCC, *TRIM2* functions as an antitumor gene as well as a specific prognostic indicator, and its exogenous overexpression suppresses cell proliferation, migration, and invasion (29). *TRIM26* functions as a tumor suppressor in hepatocellular carcinoma, papillary thyroid carcinoma, and non-small cell lung cancer (40–42), but its role in KIRC awaits future exploration. The expression of *TRIM27* and *TRIM47* was remarkably higher in KIRC tumor compared to that in normal tissues, and their increased expression was associated with worse OS, indicating an oncogenic role in KIRC. In fact, *TRIM27* is a potent oncogene in various tumor types. Silencing or genetic knockout of *TRIM27* ubiquitination-dependently or -independently inhibits malignant biological behavior in breast, colorectal, and ovarian cancers (43–45). The expression of *TRIM27* in RCC is a specific prognostic indicator, and its overexpression accelerates cell proliferation by promoting IκBα ubiquitination and inducing the activation of NF-κB signaling (46). Although more *TRIM13*, *35*, and *55* mRNA was expressed in KIRC tumor tissues, this might favor a better clinical prognosis for patients with KIRC. Therefore, the increased expression of *TRIM13*, *35*, and *55* in KIRC tissues was inconsistent with their predictive performance in KIRC. Levels of TRIM13 protein are downregulated in ccRCC and associated with a short OS (47). The forced expression of TRIM13 reduces cell proliferation, migration, and invasion. *TRIM35* is a tumor suppressor in HCC but suppresses HCC cell tumorigenicity by blocking PKM2 phosphorylation (48). *TRIM35* plays a vital role in the tumoral growth of lung cancer and might be a potential diagnostic and prognostic target for patients with lung cancer (49). Thus, *TRIM35* exerts both oncogenic and tumor suppressor roles in diverse types of tumors and its role in KIRC requires further exploration. Abundant *TRIM55* is expressed in cardiac and skeletal muscles and plays significant roles in cardiomyocyte hypertrophy and apoptosis (50, 51). The forced expression of TRIM55 inhibits hepatocellular carcinoma cell migration and invasion by reversing the epithelial to mesenchymal transition (52). However, its role in KIRC and other types of tumors remains unknown and awaits further investigation.

Our study has some limitations. Our analysis was based on retrospective cohorts from public databases. Therefore, validation of the TRIM-based signature in prospective cohorts is required. The sample size used to verify the expression of the eight TRIM genes is a little small and the expression of eight TRIM genes should also be detected in non-neoplastic kidney pathologies. The functional roles of the eight prognostic TRIM genes in KIRC require further exploration *in vitro* and *in vivo*.

In conclusion, we constructed a novel gene signature of the TRIM family in KIRC and showed that it could predict the prognosis of patients with KIRC. Furthermore, our prognostic signature is associated with tumor immune status and distinct immune cell infiltrates in the tumor microenvironment.

## Data Availability Statement

The datasets presented in this study can be found in online repositories. The names of the repository/repositories and accession number(s) can be found in the article/**Supplementary Material**.

## Ethics Statement

The studies involving human participants were reviewed and approved by Ethics Committee of Renmin Hospital of Wuhan University. The patients/participants provided their written informed consent to participate in this study.

## Author Contributions

FC designed the study. DZ conducted bioinformatic analysis with the help from YZ and YX. DZ wrote the manuscript and responsible for language revisions. All authors reviewed the manuscript. All authors contributed to the article and approved the submitted version.

## Funding

This study was supported by grants from National Natural Science Foundation of China (81870471 and 81800617) and Science and Technology Major Project of Hubei Province (2019AEA170).

## Conflict of Interest

The authors declare that the research was conducted in the absence of any commercial or financial relationships that could be construed as a potential conflict of interest.

## Publisher’s Note

All claims expressed in this article are solely those of the authors and do not necessarily represent those of their affiliated organizations, or those of the publisher, the editors and the reviewers. Any product that may be evaluated in this article, or claim that may be made by its manufacturer, is not guaranteed or endorsed by the publisher.

## References

[1] Joosten SC Smits KM Aarts MJ Melotte V Koch A Tjan-Heijnen VC. Epigenetics in Renal Cell Cancer: Mechanisms and Clinical Applications. Nat Rev Urol (2018) 15:430–51. doi: 10.1038/s41585-018-0023-z 29867106

[2] Capitanio U Bensalah K Bex A Boorjian SA Bray F Coleman J. Epidemiology of Renal Cell Carcinoma. Eur Urol (2019) 75:74–84. doi: 10.1016/j.eururo.2018.08.036 30243799 PMC8397918

[3] Xiao Y Meierhofer D. Glutathione Metabolism in Renal Cell Carcinoma Progression and Implications for Therapies. Int J Mol Sci (2019) 20:3672. doi: 10.3390/ijms20153672 PMC6696504 31357507

[4] Zuo S Wang L Wen Y Dai G. Identification of a Universal 6-lncRNA Prognostic Signature for Three Pathologic Subtypes of Renal Cell Carcinoma. J Cell Biochem (2018) 120:7375–85. doi: 10.1002/jcb.28012 30378181

[5] Posadas EM Limvorasak S Figlin RA. Targeted Therapies for Renal Cell Carcinoma. Nat Rev Nephrol (2017) 13:496–511. doi: 10.1038/nrneph.2017.82 28691713

[6] Hsieh JJ Purdue MP Signoretti S Swanton C Albiges L Schmidinger M. Renal Cell Carcinoma. Nat Rev Dis Primers (2017) 3:17009. doi: 10.1038/nrdp.2017.9 28276433 PMC5936048

[7] Hatakeyama S. TRIM Family Proteins: Roles in Autophagy, Immunity, and Carcinogenesis. Trends Biochem Sci (2017) 42:297–311. doi: 10.1016/j.tibs.2017.01.002 28118948

[8] Esposito D Koliopoulos MG Rittinger K. Structural Determinants of TRIM Protein Function. Biochem Soc Trans (2017) 45:183–91. doi: 10.1042/bst20160325 28202672

[9] Ozato K Shin DM Chang TH Morse HC 3rd. TRIM Family Proteins and Their Emerging Roles in Innate Immunity. Nat Rev Immunol (2008) 8:849–60. doi: 10.1038/nri2413 PMC3433745 18836477

[10] Alloush J Weisleder N. TRIM Proteins in Therapeutic Membrane Repair of Muscular Dystrophy. JAMA Neurol (2013) 70:928–31. doi: 10.1001/jamaneurol.2013.469 PMC3746993 23699904

[11] Liu J Zhang C Wang X Hu W Feng Z. Tumor Suppressor P53 Cross-Talks With TRIM Family Proteins. Genes Dis (2021) 8:463–74. doi: 10.1016/j.gendis.2020.07.003 PMC8209353 34179310

[12] Pauletto E Eickhoff N Padrão NA Blattner C Zwart W. TRIMming Down Hormone-Driven Cancers: The Biological Impact of TRIM Proteins on Tumor Development, Progression and Prognostication. Cells (2021) 10:1517. doi: 10.3390/cells10061517 34208621 PMC8234875

[13] Bhaduri U Merla G. Rise of TRIM8: A Molecule of Duality. Mol Ther Nucleic Acids (2020) 22:434–44. doi: 10.1016/j.omtn.2020.08.034 PMC7533350 33230447

[14] Chen JX Xu D Cao JW Zuo L Han ZT Tian YJ. TRIM47 Promotes Malignant Progression of Renal Cell Carcinoma by Degrading P53 Through Ubiquitination. Cancer Cell Int (2021) 21:129. doi: 10.1186/s12935-021-01831-0 33622324 PMC7903798

[15] Kamps R Brandão RD Bosch BJ Paulussen AD Xanthoulea S Blok MJ. Next-Generation Sequencing in Oncology: Genetic Diagnosis, Risk Prediction and Cancer Classification. Int J Mol Sci (2017) 18:308. doi: 10.3390/ijms18020308 PMC5343844 28146134

[16] Yohe S Thyagarajan B. Review of Clinical Next-Generation Sequencing. Arch Pathol Lab Med (2017) 141:1544–57. doi: 10.5858/arpa.2016-0501-RA 28782984

[17] Lv Z Qi L Hu X Mo M Jiang H Li Y. Identification of a Novel Glycolysis-Related Gene Signature Correlates With the Prognosis and Therapeutic Responses in Patients With Clear Cell Renal Cell Carcinoma. Front Oncol (2021) 11:633950. doi: 10.3389/fonc.2021.633950 33816274 PMC8010189

[18] Wu Y Zhang X Wei X Feng H Hu B Deng Z. Development of an Individualized Ubiquitin Prognostic Signature for Clear Cell Renal Cell Carcinoma. Front Cell Dev Biol (2021) 9:684643. doi: 10.3389/fcell.2021.684643 34239875 PMC8258262

[19] Beuselinck B Job S Becht E Karadimou A Verkarre V Couchy G. Molecular Subtypes of Clear Cell Renal Cell Carcinoma are Associated With Sunitinib Response in the Metastatic Setting. Clin Cancer Res (2015) 21:1329–39. doi: 10.1158/1078-0432.Ccr-14-1128 25583177

[20] Verbiest A Couchy G Job S Zucman-Rossi J Caruana L Lerut E. Molecular Subtypes of Clear Cell Renal Cell Carcinoma Are Associated With Outcome During Pazopanib Therapy in the Metastatic Setting. Clin Genitourin Cancer (2018) 16:e605–12. doi: 10.1016/j.clgc.2017.10.017 29239846

[21] Verbiest A Renders I Caruso S Couchy G Job S Laenen A. Clear-Cell Renal Cell Carcinoma: Molecular Characterization of IMDC Risk Groups and Sarcomatoid Tumors. Clin Genitourin Cancer (2019) 17:e981–94. doi: 10.1016/j.clgc.2019.05.009 31229459

[22] Jaworska AM Wlodarczyk NA Mackiewicz A Czerwinska P. The Role of TRIM Family Proteins in the Regulation of Cancer Stem Cell Self-Renewal. Stem Cells (2020) 38:165–73. doi: 10.1002/stem.3109 PMC7027504 31664748

[23] Wu G Xu Y Li L Li J Ruan N Dong J. Tripartite-Motif Family Genes Associated With Cancer Stem Cells Affect Tumor Progression and can Assist in the Clinical Prognosis of Kidney Renal Clear Cell Carcinoma. Int J Med Sci (2020) 17:2905–16. doi: 10.7150/ijms.51260 PMC7646106 33173411

[24] Zhang L Afolabi LO Wan X Li Y Chen L. Emerging Roles of Tripartite Motif-Containing Family Proteins (TRIMs) in Eliminating Misfolded Proteins. Front Cell Dev Biol (2020) 8:802. doi: 10.3389/fcell.2020.00802 32984318 PMC7479839

[25] Zhan W Zhang S. TRIM Proteins in Lung Cancer: Mechanisms, Biomarkers and Therapeutic Targets. Life Sci (2021) 268:118985. doi: 10.1016/j.lfs.2020.118985 33412211

[26] Mastropasqua F Marzano F Valletti A Aiello I Di Tullio G Morgano A. TRIM8 Restores P53 Tumour Suppressor Function by Blunting N-MYC Activity in Chemo-Resistant Tumours. Mol Cancer (2017) 16:67. doi: 10.1186/s12943-017-0634-7 28327152 PMC5359838

[27] Zhang C Mukherjee S Tucker-Burden C Ross JL Chau MJ Kong J. TRIM8 Regulates Stemness in Glioblastoma Through PIAS3-Stat3. Mol Oncol (2017) 11:280–94. doi: 10.1002/1878-0261.12034 PMC5332279 28100038

[28] Li X Yuan J Song C Lei Y Xu J Zhang G. Deubiquitinase USP39 and E3 Ligase TRIM26 Balance the Level of ZEB1 Ubiquitination and Thereby Determine the Progression of Hepatocellular Carcinoma. Cell Death Differ (2021) 28:2315–32. doi: 10.1038/s41418-021-00754-7 PMC8329202 33649471

[29] Xiao W Wang X Wang T Xing J. TRIM2 Downregulation in Clear Cell Renal Cell Carcinoma Affects Cell Proliferation, Migration, and Invasion and Predicts Poor Patients' Survival. Cancer Manag Res (2018) 10:5951–64. doi: 10.2147/cmar.S185270 PMC6255054 30538545

[30] Yamada Y Kimura N Takayama KI Sato Y Suzuki T Azuma K. TRIM44 Promotes Cell Proliferation and Migration by Inhibiting FRK in Renal Cell Carcinoma. Cancer Sci (2020) 111:881–90. doi: 10.1111/cas.14295 PMC7060480 31883420

[31] Miao C Liang C Li P Liu B Qin C Yuan H. TRIM37 Orchestrates Renal Cell Carcinoma Progression via Histone H2A Ubiquitination-Dependent Manner. J Exp Clin Cancer Res (2021) 40:195. doi: 10.1186/s13046-021-01980-0 34130705 PMC8204444

[32] Helmink BA Reddy SM Gao J Zhang S Basar R Thakur R. B Cells and Tertiary Lymphoid Structures Promote Immunotherapy Response. Nature (2020) 577:549–55. doi: 10.1038/s41586-019-1922-8 PMC8762581 31942075

[33] Giraldo NA Becht E Vano Y Petitprez F Lacroix L Validire P. Tumor-Infiltrating and Peripheral Blood T-Cell Immunophenotypes Predict Early Relapse in Localized Clear Cell Renal Cell Carcinoma. Clin Cancer Res (2017) 23:4416–28. doi: 10.1158/1078-0432.Ccr-16-2848 28213366

[34] Zhang S Zhang E Long J Hu Z Peng J Liu L. Immune Infiltration in Renal Cell Carcinoma. Cancer Sci (2019) 110:1564–72. doi: 10.1111/cas.13996 PMC6501001 30861269

[35] Obad S Olofsson T Mechti N Gullberg U Drott K. Regulation of the Interferon-Inducible P53 Target Gene TRIM22 (Staf50) in Human T Lymphocyte Activation. J Interferon Cytokine Res (2007) 27:857–64. doi: 10.1089/jir.2006.0180 17970695

[36] Wei J Guo C An X Miao W Zhang C Wang B. Tumor Cell-Expressed IL-15rα Drives Antagonistic Effects on the Progression and Immune Control of Gastric Cancer and is Epigenetically Regulated in EBV-Positive Gastric Cancer. Cell Oncol (Dordr) (2020) 43:1085–97. doi: 10.1007/s13402-020-00542-4 PMC12990744 32767257

[37] Qin Y Ye J Zhao F Hu S Wang S. TRIM2 Regulates the Development and Metastasis of Tumorous Cells of Osteosarcoma. Int J Oncol (2018) 53:1643–56. doi: 10.3892/ijo.2018.4494 30066883

[38] Cao H Fang Y Liang Q Wang J Luo B Zeng G. TRIM2 is a Novel Promoter of Human Colorectal Cancer. Scand J Gastroenterol (2019) 54:210–8. doi: 10.1080/00365521.2019.1575463 30916596

[39] Sun Q Ye Z Qin Y Fan G Ji S Zhuo Q. Oncogenic Function of TRIM2 in Pancreatic Cancer by Activating ROS-Related NRF2/ITGB7/FAK Axis. Oncogene (2020) 39:6572–88. doi: 10.1038/s41388-020-01452-3 32929153

[40] Wang Y He D Yang L Wen B Dai J Zhang Q. TRIM26 Functions as a Novel Tumor Suppressor of Hepatocellular Carcinoma and its Downregulation Contributes to Worse Prognosis. Biochem Biophys Res Commun (2015) 463:458–65. doi: 10.1016/j.bbrc.2015.05.117 26043685

[41] Wang K Chai L Qiu Z Zhang Y Gao H Zhang X. Overexpression of TRIM26 Suppresses the Proliferation, Metastasis, and Glycolysis in Papillary Thyroid Carcinoma Cells. J Cell Physiol (2019) 234:19019–27. doi: 10.1002/jcp.28541 30927273

[42] Tao JL Luo M Sun H Zhao HM Sun QS Huang ZM. Overexpression of Tripartite Motif Containing 26 Inhibits Non-Small Cell Lung Cancer Cell Growth by Suppressing PI3K/AKT Signaling. Kaohsiung J Med Sci (2020) 36:417–22. doi: 10.1002/kjm2.12194 PMC11896303 32052576

[43] Ma Y Wei Z Bast RC Jr Wang Z Li Y Gao M. Downregulation of TRIM27 Expression Inhibits the Proliferation of Ovarian Cancer Cells *In Vitro* and *In Vivo* . Lab Invest (2016) 96:37–48. doi: 10.1038/labinvest.2015.132 26568293

[44] Zhang Y Feng Y Ji D Wang Q Qian W Wang S. TRIM27 Functions as an Oncogene by Activating Epithelial-Mesenchymal Transition and P-AKT in Colorectal Cancer. Int J Oncol (2018) 53:620–32. doi: 10.3892/ijo.2018.4408 PMC6017157 29767249

[45] Xing L Tang X Wu K Huang X Yi Y Huan J. TRIM27 Functions as a Novel Oncogene in Non-Triple-Negative Breast Cancer by Blocking Cellular Senescence Through P21 Ubiquitination. Mol Ther Nucleic Acids (2020) 22:910–23. doi: 10.1016/j.omtn.2020.10.012 PMC7666371 33251042

[46] Xiao C Zhang W Hua M Chen H Yang B Wang Y. TRIM27 Interacts With Iκbα to Promote the Growth of Human Renal Cancer Cells Through Regulating the NF-κb Pathway. BMC Cancer (2021) 21:841. doi: 10.1186/s12885-021-08562-5 34284744 PMC8293539

[47] Li H Qu L Zhou R Wu Y Zhou S Zhang Y. TRIM13 Inhibits Cell Migration and Invasion in Clear-Cell Renal Cell Carcinoma. Nutr Cancer (2020) 72:1115–24. doi: 10.1080/01635581.2019.1675721 31762344

[48] Chen Z Wang Z Guo W Zhang Z Zhao F Zhao Y. TRIM35 Interacts With Pyruvate Kinase Isoform M2 to Suppress the Warburg Effect and Tumorigenicity in Hepatocellular Carcinoma. Oncogene (2015) 34:3946–56. doi: 10.1038/onc.2014.325 25263439

[49] Zhang J Xu Z Yu B Xu J Yu B. Tripartite Motif Containing 35 Contributes to the Proliferation, Migration, and Invasion of Lung Cancer Cells *In Vitro* and *In Vivo* . Biosci Rep (2020) 40:BSR20200065. doi: 10.1042/bsr20200065 32293015 PMC7198043

[50] Heliste J Chheda H Paatero I Salminen TA Akimov Y Paavola J. Genetic and Functional Implications of an Exonic TRIM55 Variant in Heart Failure. J Mol Cell Cardiol (2020) 138:222–33. doi: 10.1016/j.yjmcc.2019.12.008 31866377

[51] Tan J Shen J Zhu H Gong Y Zhu H Li J. miR-378a-3p Inhibits Ischemia/Reperfusion-Induced Apoptosis in H9C2 Cardiomyocytes by Targeting TRIM55 via the DUSP1-JNK1/2 Signaling Pathway. Aging (Albany NY) (2020) 12:8939–52. doi: 10.18632/aging.103106 PMC7288954 32463795

[52] Li X Huang L Gao W. Overexpression of Tripartite Motif Conaining 55 (TRIM55) Inhibits Migration and Invasion of Hepatocellular Carcinoma (HCC) Cells *via* Epithelial-Mesenchymal Transition and Matrix Metalloproteinase-2 (Mmp2). Med Sci Monit (2019) 25:771–7. doi: 10.12659/msm.910984 PMC6360872 30685767

