# Supplementary material for: A Novel Gene Signature of Tripartite Motif Family for Predicting the Prognosis in Kidney Renal Clear Cell Carcinoma and Its Association With Immune Cell Infiltration
Source: Front Oncol. 2022 Mar 17;12:840410. doi: 10.3389/fonc.2022.840410 (PMC8968921; doi:10.3389/fonc.2022.840410)
Supplement: Supplementary file 1 [file DataSheet_1.docx]

Supplementary Material


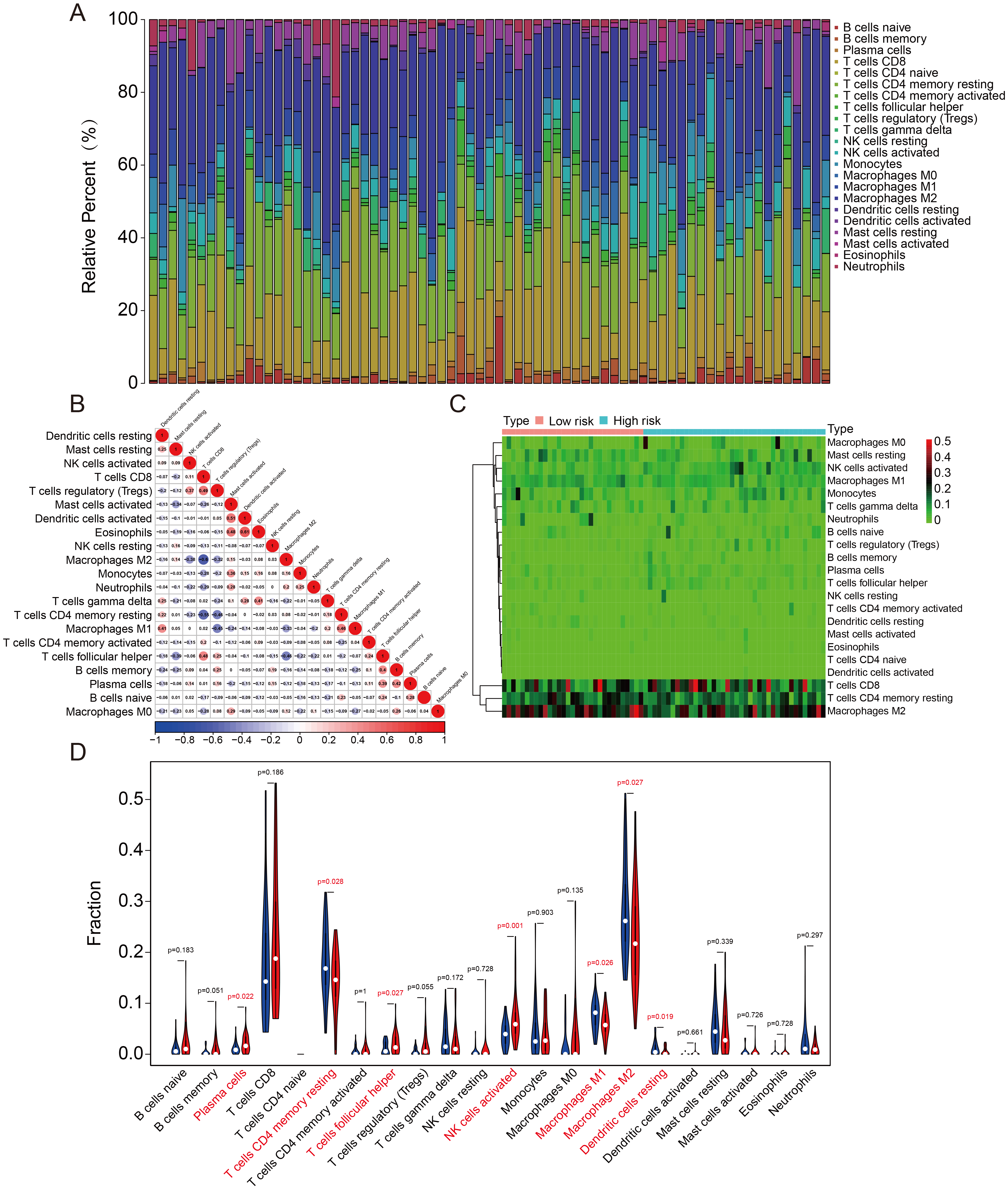


**Supplementary Figure 1.** The eight-gene signature of TRIM family was correlated with tumor immune cell infiltration in ICGC KIRC cohort. (A) Stacked bar chart showing the abundance of 22 immune cell types in each KIRC sample of the ICGC cohort. (B) The correlation heatmap of the infiltrating immune cells in the ICGC cohort. (C-D) Heatmap and violin plot exhibiting immune cell infiltrates in KIRC patients at high- and low-risk groups.
